# Nano-SiO_2_/Polymer Composite Gel Fluid-Loss Reducer for High-Temperature, Salt-Resistant Water-Based Drilling Fluids: Performance and Mechanism

**DOI:** 10.3390/gels12100895

**Published:** 2026-10-03

**Authors:** Luman Liu, Jingping Liu, Yuan Geng, Ren Wang, Beiqiao Meng, Shifeng Gao, He Li, Zhe Xu, Yi An, Yuanwei Sun

**Affiliations:** 1 CNPC Engineering Technology R&D Co. Ltd., Beijing 102206, China; 2 School of Petroleum Engineering, China University of Petroleum—East China (UPC), Qingdao 266580, China; 3 Project Department of Tight Oil and Gas Exploration and Development, PetroChina Southwest Oil & Gas Field Company, Chengdu 610051, China

**Keywords:** nanocomposite fluid-loss reducer, water-based drilling fluid, high-temperature resistance, salt resistance

## Abstract

Polymeric gel fluid-loss reducers for water-based drilling fluids often undergo performance deterioration under combined high-temperature and high-salinity conditions. In this study, an organic–inorganic nanocomposite gel fluid-loss reducer, SiO_2_@P-DAS, was synthesized through aqueous free-radical polymerization using N,N-dimethylacrylamide, 2-acrylamido-2-methylpropane sulfonic acid, sodium styrene sulfonate, and KH-570-modified nano-SiO_2_. The corresponding polymer gel without nano-SiO_2_, P-DAS, was used as the control. FTIR results were consistent with the presence of modified nano-SiO_2_ in the composite gel. Thermogravimetric analysis showed that its initial decomposition temperature increased from 245 °C for P-DAS to 252 °C for SiO_2_@P-DAS. At a concentration of 3.0 wt%, the post-aging API filtrate volume of SiO_2_@P-DAS was 3.8 mL, compared with 4.8 mL for P-DAS after aging at 220 °C for 16 h. Under HTHP conditions of 220 °C and 3.5 MPa, the corresponding filtrate volumes were 33.33 and 49.67 mL, respectively. SiO_2_@P-DAS also maintained lower fluid loss under NaCl and CaCl_2_ contamination. At 5 wt% CaCl_2_, its post-aging API filtrate volume was 6.8 mL, 58.0% lower than that of P-DAS. In weighted drilling fluids with densities of 1.4–2.0 g/cm^3^, the post-aging API and HTHP filtrate volumes remained within 2.4–3.2 and 24.6–26.2 mL, respectively. Zeta-potential, particle-size, and filter-cake observations suggest that polymer adsorption and hydration, together with the contribution of modified nano-SiO_2_ to particle packing and filter-cake continuity, improve filtration control. These results demonstrate the potential of SiO_2_@P-DAS as a nanocomposite gel fluid-loss reducer for high-temperature and salt-contaminated water-based drilling fluids.

## 1. Introduction

Drilling deep hydrocarbon reservoirs commonly involves complex downhole conditions, including temperatures above 200 °C, contamination from salt-gypsum formations, and low-quality clay solids [1,2]. Drilling fluids perform essential functions such as transporting cuttings, balancing formation pressure, cooling and lubricating the drillstring, stabilizing the wellbore, and protecting hydrocarbon-bearing formations; consequently, their stability directly affects drilling safety and efficiency. Compared with oil-based drilling fluids, water-based drilling fluids offer lower cost, better environmental compatibility, and easier maintenance. Under high-temperature and high-salinity conditions, however, their colloidal stability and rheological properties can change markedly. Elevated temperature weakens the adsorption of treatment agents on clay surfaces and may degrade their molecular structures. Meanwhile, high electrolyte concentrations compress the diffuse electrical double layer around clay particles, disrupt hydration films, and induce aggregation. The resulting filter cake is loose, with greater pore connectivity and permeability, which ultimately increases fluid loss. Maintaining the rheological stability and filtration-control performance of water-based drilling fluids under high-temperature, high-salinity conditions therefore remains a major challenge in deep and ultradeep drilling.

Fluid-loss reducers control filtration by adsorbing onto and encapsulating clay particles, improving particle-size gradation, and reducing filter-cake permeability; they are therefore key additives in high-temperature, high-salinity water-based drilling fluids [3,4,5,6]. Current research focuses mainly on polymeric materials. Hydrolysis-resistant, strongly hydrophilic, and structurally rigid functional monomers are selected to tailor polymer-chain architecture and functional-group composition, thereby improving thermal stability, brine compatibility, and adsorption-based protection of clay particles. DMAA, AMPS, and SSS provide complementary structural functions. DMAA contributes hydrophilic nonionic amide groups, and its N-methyl-substituted structure improves hydrolytic stability. AMPS hydrates strongly and remains ionized over a broad pH range. SSS combines the rigidity of an aromatic ring with the hydration characteristics of a sulfonate group, which benefits backbone thermal stability and brine compatibility. Copolymerization of these three monomers is therefore expected to improve temperature resistance, salt tolerance, and adsorption onto clay [7,8,9,10,11]. Previous studies have shown that DMAA/AMPS binary copolymers improve temperature and salt resistance but may still provide inadequate filtration control in high-temperature saturated brines. Incorporating monomers such as acrylic acid enhances adsorption and hydration, although stability remains limited at 220 °C under high salinity. Increasing the fraction of sulfonated monomers can improve brine compatibility but cannot completely suppress chain contraction and clay aggregation induced by combined high temperature and salinity. Studies outside China have likewise shown that combining AMPS with cyclic or sulfonated units can markedly improve rheology and filtration performance at high temperature and mineralization, and that SSS content strongly influences fluid loss and filter-cake structure. Overall, DMAA, AMPS, and SSS offer complementary hydration, salt-resistance, and chain-rigidity properties, but further structural optimization is required to limit chain degradation and adsorption loss under extreme conditions [12,13,14].

Organic–inorganic nanocompositing is an important route for improving the temperature and salt resistance of polymeric fluid-loss reducers. Nano-SiO_2_ has a high specific surface area and a stable siloxane framework. Its rigid inorganic structure can restrict thermal motion of polymer segments, while the nanoparticles can enter filter-cake pores and improve particle packing, thereby reducing porosity and permeability [15,16]. Unmodified nano-SiO_2_, however, readily aggregates because of its high surface energy and compression of the electrical double layer in concentrated brines, which undermines long-term stability and reinforcement. Surface functionalization with KH-570 introduces polymerizable double bonds, enabling nano-SiO_2_ to be incorporated into the polymer network through covalent bonds or as crosslinking nodes. This improves interfacial compatibility, inhibits aggregation, strengthens segmental confinement, and enhances thermal stability [17,18,19]. Covalent nanocomposites based on silanized nano-SiO_2_ have been reported to retain structure more effectively at high temperature than physical blends and to form denser, lower-permeability filter cakes through the combined effects of nanoscale filling and polymeric colloid protection [20,21]. Modified nano-SiO_2_ in core–shell or grafted structures can also stabilize chain conformation in concentrated brines and strengthen the network between hydrated bentonite particles [22,23,24,25,26,27]. Thus, surface-functionalized nano-SiO_2_ provides a promising combination of inorganic reinforcement, polymer adsorption, and nanoscale plugging for high-temperature, salt-resistant fluid-loss reducers [28,29,30,31,32].

Although previous studies have demonstrated the potential of polymer/nano-SiO_2_ composites as high-temperature and salt-resistant fluid-loss reducers, the specific contribution of modified nano-SiO_2_ is often difficult to distinguish because the polymer composition, nanoparticle type, preparation procedure, and evaluation conditions vary among studies. Consequently, a directly matched comparison using the same polymer composition and synthesis conditions is needed to determine whether the incorporation of modified nano-SiO_2_ provides a measurable improvement over the corresponding nano-free polymer under combined high-temperature and high-salinity conditions. To address this issue, P-DAS and SiO_2_@P-DAS were prepared using the same DMAA/AMPS/SSS composition and synthesis parameters, with the incorporation of KH-570-modified nano-SiO_2_ as the principal structural difference. Their thermal stability, rheological behavior, API filtration performance, and NaCl resistance were systematically compared. Zeta-potential, particle-size, and filter-cake morphology analyses were further used to examine the relationship between nano-SiO_2_ incorporation, particle dispersion, and filtration control. This matched comparative design provides an experimental basis for evaluating the role of modified nano-SiO_2_ in DMAA/AMPS/SSS-based fluid-loss reducers.

## 2. Results and Discussion

### 2.1. Characterization

#### 2.1.1. FTIR Analysis

**Figure 1:**
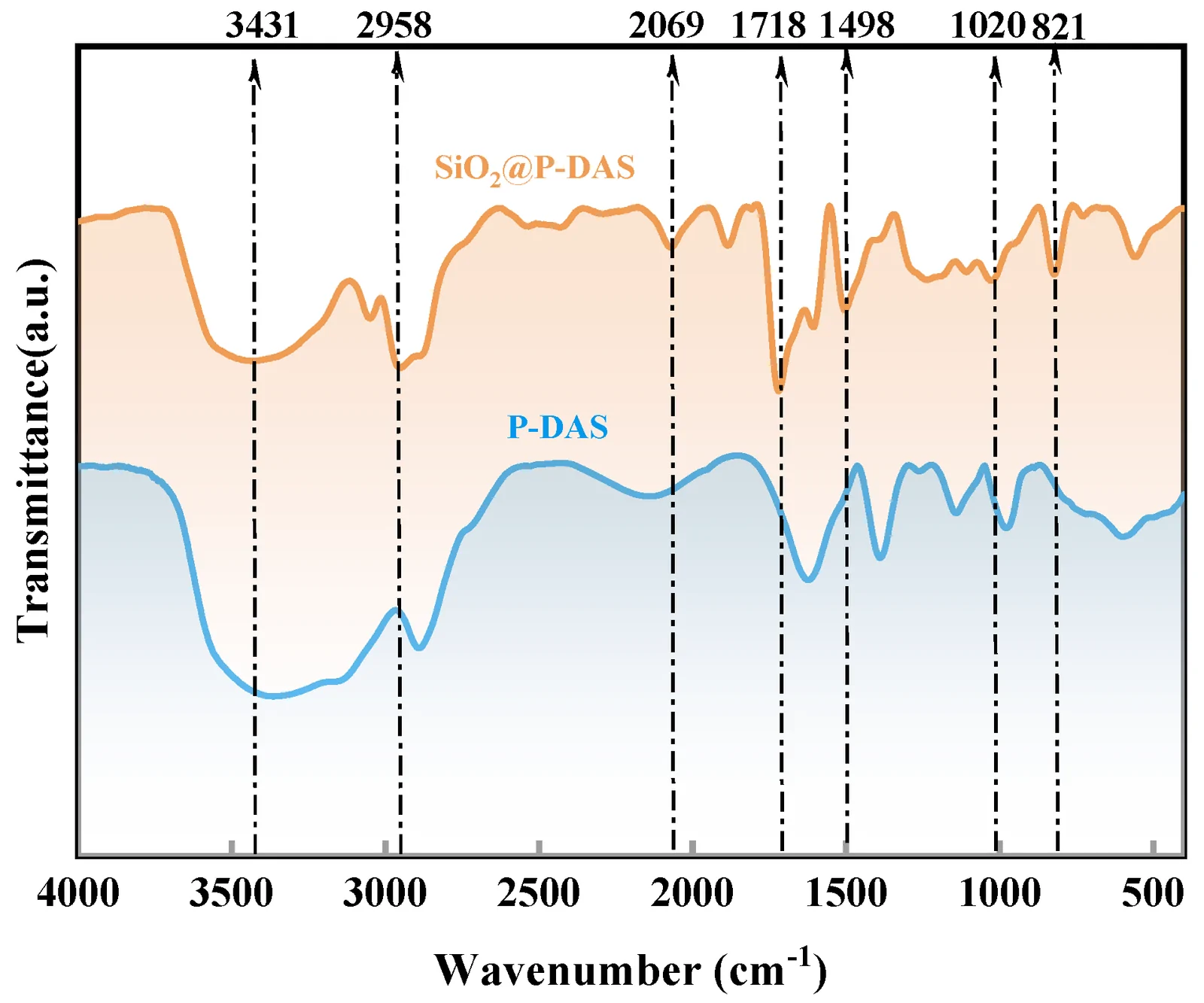
FTIR spectra of SiO_2_@P-DAS and P-DAS.

The FTIR spectra of SiO_2_@P-DAS and P-DAS are shown in Figure 1. The P-DAS spectrum exhibits a vibration band of SO_3_^−^ at 1027 cm^−1^, a C-O bending band at 1196 cm^−1^, a C-N stretching band at 1442 cm^−1^, a -CH_2_- stretching band at 2930 cm^−1^, and an N-H stretching band at 3395 cm^−1^, confirming successful synthesis of the DMAA/AMPS/SSS terpolymer. SiO_2_@P-DAS retains all characteristic signals of the terpolymer. The band at 3431 cm^−1^ is assigned to overlapping stretching vibrations of amide N-H, silanol Si-OH, and O-H in adsorbed water; 2958 cm^−1^ corresponds to saturated C-H stretching in the main chain; 1718 cm^−1^ is attributed to amide C=O stretching; 1498 cm^−1^ arises from overlapping C-H bending and aromatic-ring skeletal vibrations of SSS; 1020 cm^−1^ contains contributions from S=O stretching of sulfonate groups and asymmetric Si-O-Si stretching; and 821 cm^−1^ is characteristic of Si-O vibration. No C=C absorption band was observed, indicating that the monomer double bonds participated fully in copolymerization. SiO_2_@P-DAS retained the characteristic absorption bands of the P-DAS framework and exhibited additional signals associated with siloxane groups. These spectral features are consistent with the presence of KH-570-modified nano-SiO_2_ in the resulting composite.

#### 2.1.2. Thermogravimetric Analysis

**Figure 2:**
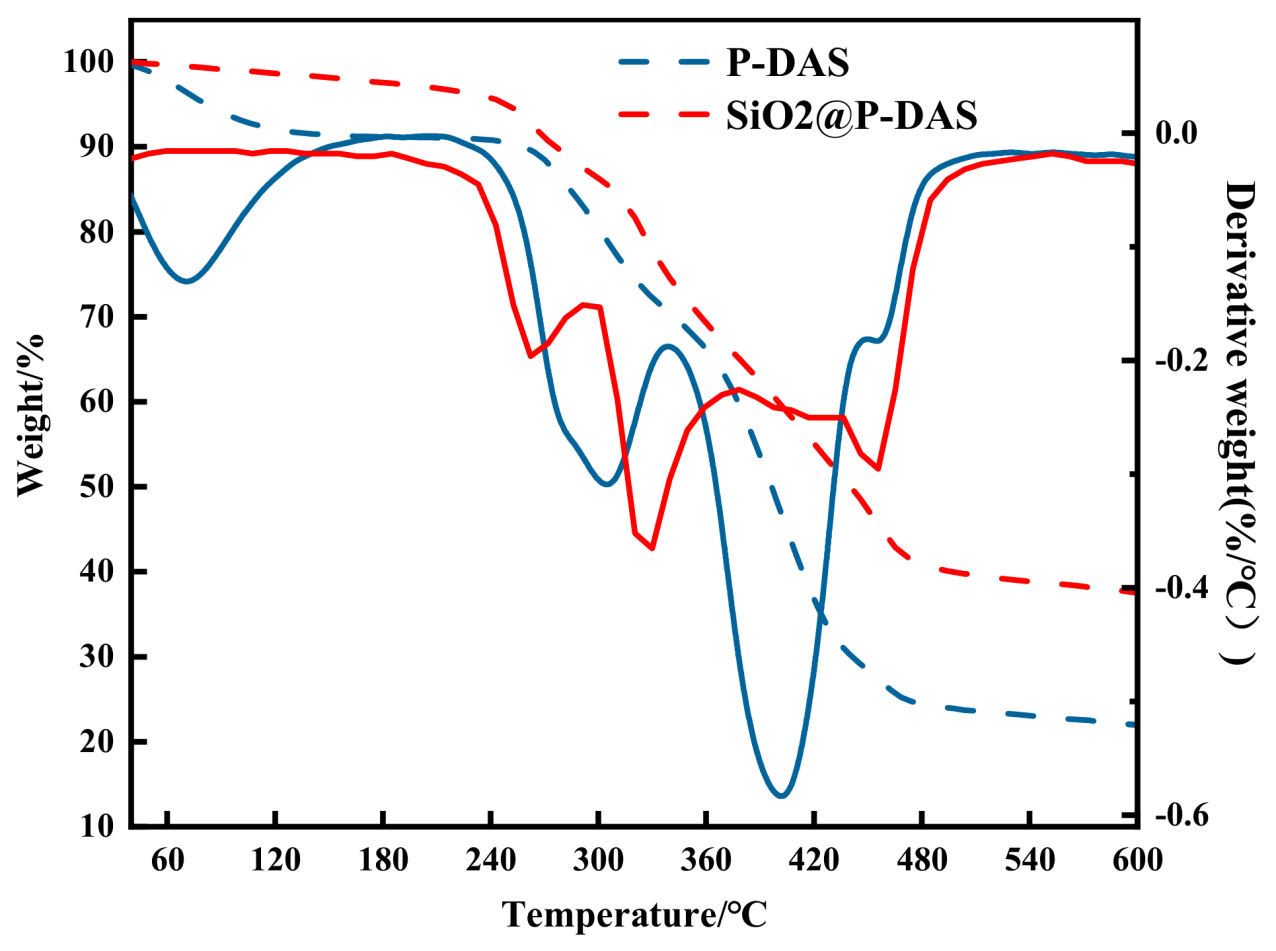
Thermogravimetric curves of SiO_2_@P-DAS and P-DAS.

As shown in Figure 2, P-DAS and SiO_2_@P-DAS both exhibit the typical three-stage mass-loss behavior. The first stage primarily corresponds to removal of adsorbed and bound water: P-DAS lost 9.8% between 25 and 245 °C, whereas SiO_2_@P-DAS lost 4.4% between 40 and 252 °C. The second stage is the principal thermal-decomposition stage, during which side-chain functional groups and the polymer backbone decompose. P-DAS lost 59.8% between 245 and 435 °C, while SiO_2_@P-DAS lost 55.8% between 252 and 480 °C. The third stage corresponds mainly to slow decomposition of the residual framework; the residual mass of SiO_2_@P-DAS at 600 °C was 8.5%. Taking the onset of the main decomposition stage as the initial decomposition temperature, the values for P-DAS and SiO_2_@P-DAS were 245 and 252 °C, respectively. Incorporation of nano-SiO2 therefore raised the initial decomposition temperature by 7 °C and shifted the main decomposition range toward higher temperatures. These results indicate that the incorporation of modified nano-SiO_2_ improves the thermal stability of SiO_2_@P-DAS. The inorganic component may act as a rigid reinforcing domain that restricts the thermal motion of polymer segments and retards thermal degradation.

### 2.2. Performance Evaluation of SiO_2_@P-DAS

#### 2.2.1. Effects of SiO_2_@P-DAS on Drilling-Fluid Rheology and Filtration

**Figure 3:**
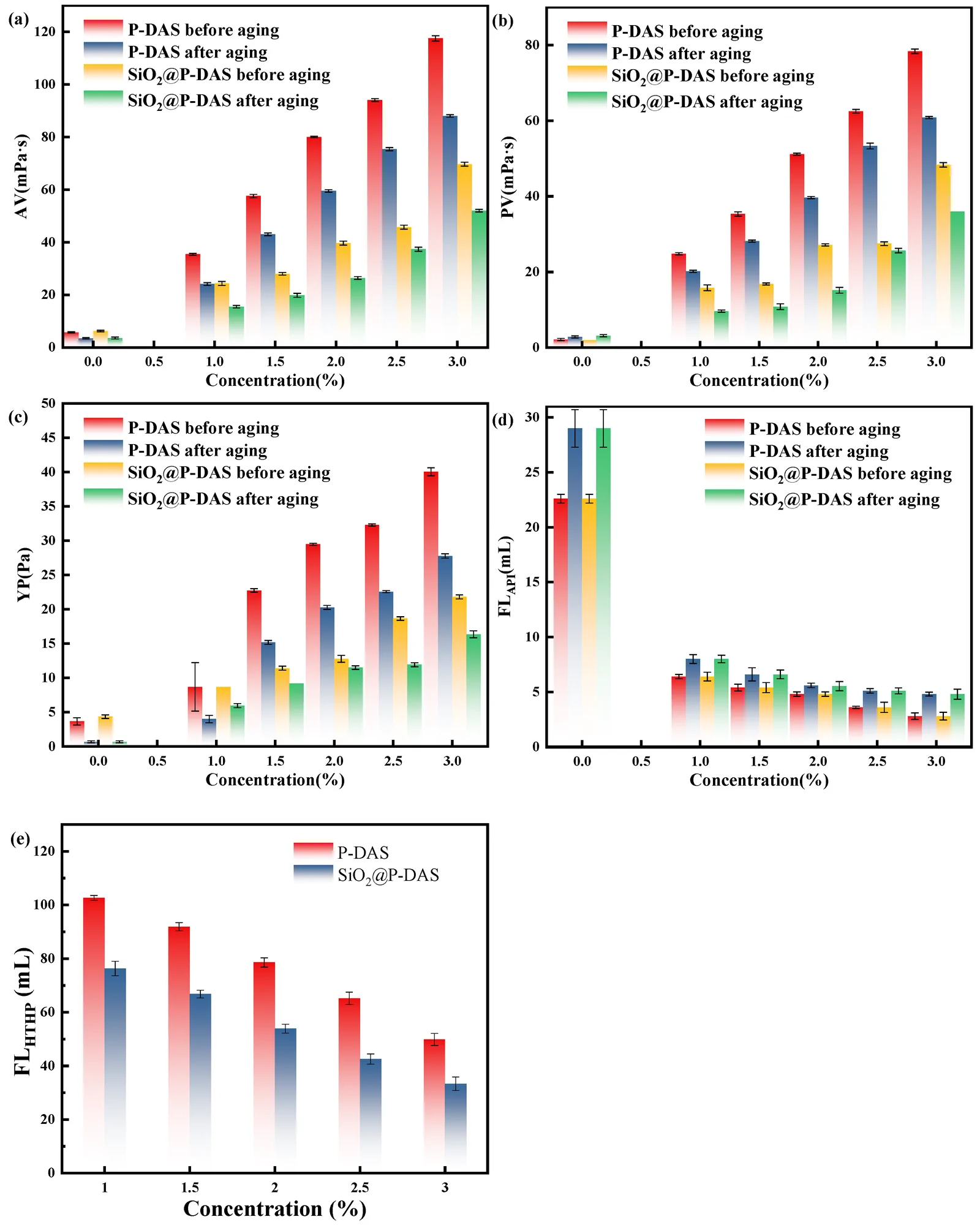
Effects of fluid-loss-reducer dosage on drilling-fluid rheology and filtration before and after aging: (**a**) AV; (**b**) PV; (**c**) YP; and (**d**) API filtrate volume. (**e**) HTHP filtrate volume.

To evaluate the effect of additive concentration and select a suitable working concentration, different concentrations of SiO_2_@P-DAS were added to a 4 wt% bentonite slurry, with P-DAS used as the control. Rheological parameters and API filtrate volumes were measured before and after aging at 220 °C for 16 h, and HTHP filtration tests were conducted at 220 °C. As shown in Figure 3, the drilling-fluid viscosity increased, whereas the API and HTHP filtrate volumes decreased with increasing additive concentration. The reduction in API filtrate volume was most pronounced at low additive concentrations and became less marked at higher concentrations. After aging, increasing the additive concentration from 0 to 1.0 wt% reduced the API filtrate volume from 29.0 to 8.0 mL for P-DAS and from 29.0 to 8.6 mL for SiO_2_@P-DAS. Increasing the concentration from 2.5 to 3.0 wt% further reduced the corresponding values from 5.1 to 4.8 mL and from 4.6 to 3.8 mL, respectively. This trend may reflect the gradual adsorption of the polymer on clay surfaces and the progressive filling of pores within the filter cake. The HTHP results further demonstrated the filtration-control advantage of SiO_2_@P-DAS. At 3.0 wt% and 220 °C, the HTHP filtrate volumes of P-DAS and SiO_2_@P-DAS were 49.67 mL and 33.33 mL, respectively, corresponding to a reduction of 32.9%. The lower API and HTHP filtrate volumes of SiO_2_@P-DAS may be attributed to the combined effects of polymer adsorption and hydration, improved particle-size distribution, and reinforcement and pore filling by modified nano-SiO_2_. Based on a comprehensive analysis of the rheological, API filtration, and HTHP filtration results, 3.0 wt% was selected as the working concentration for the subsequent temperature- and salt-resistance evaluations.

#### 2.2.2. Temperature Resistance of SiO_2_@P-DAS

**Figure 4:**
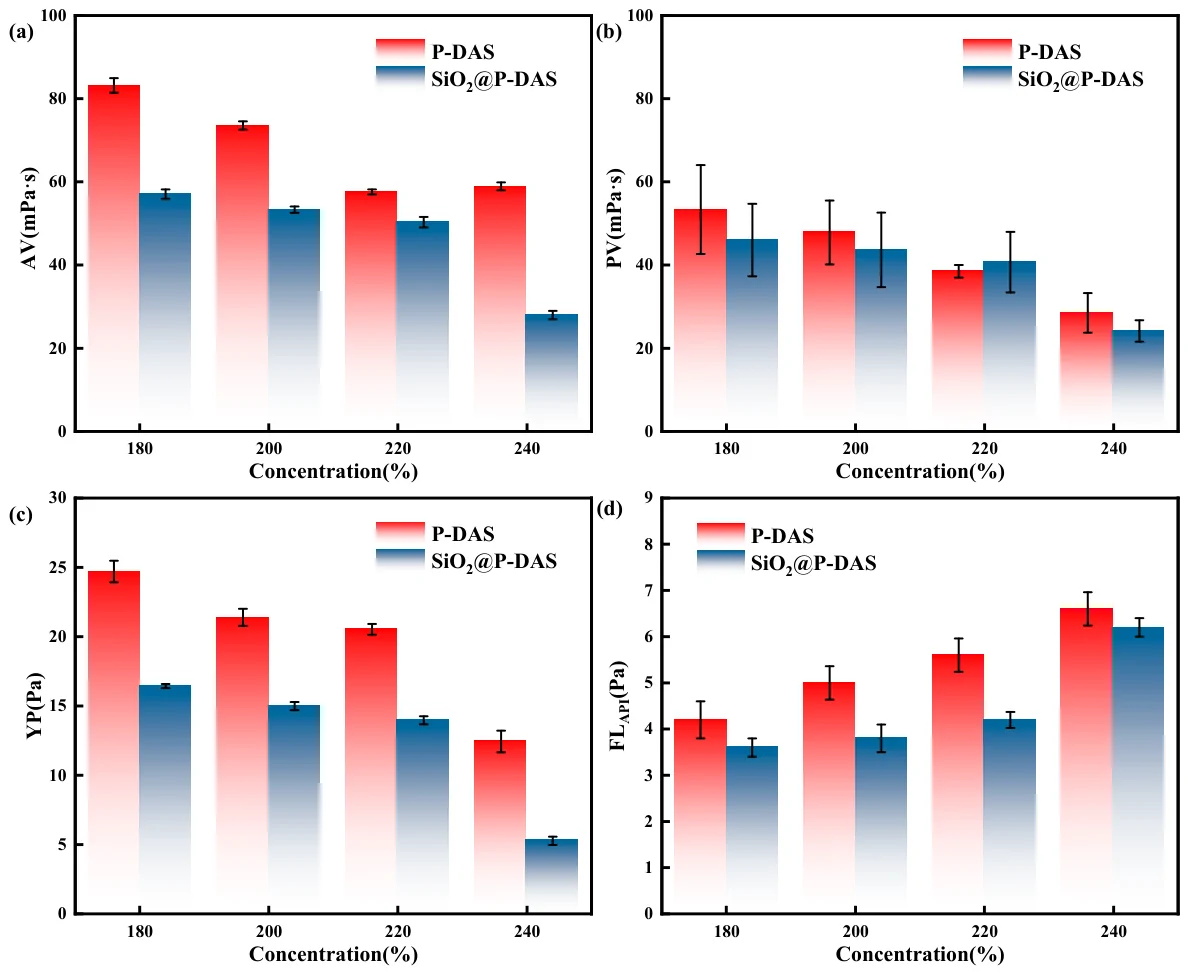
Thermal stability of P-DAS- and SiO_2_@P-DAS-treated drilling fluids after aging for 16 h at different temperatures: (**a**) AV; (**b**) PV; (**c**) YP; and (**d**) API filtrate volume.

The upper service temperature of SiO_2_@P-DAS was investigated by comparing the effects of P-DAS and SiO_2_@P-DAS on drilling-fluid properties after aging for 16 h at different temperatures. The result is shown in Figure 4. As the aging temperature increased, AV, PV, and YP of the P-DAS fluid decreased continuously, whereas API filtrate volume increased. When the temperature rose from 180 to 240 °C, AV decreased from 85 to 44 mPa s and API filtrate volume increased from 4.2 to 6.6 mL. For SiO_2_@P-DAS, increasing the temperature from 180 to 220 °C reduced AV only slightly, from 52 to 50.5 mPa s, while API filtrate volume increased from 3.6 to 4.2 mL. The modified nano-SiO_2_ introduced during polymerization may provide rigid reinforcement, restrict polymer-segment motion, and contribute to the filling of small pores in the filter cake, while the polymer component adsorbs on clay surfaces and helps maintain the colloidal structure. Above 220 °C, performance changed abruptly. At 240 °C, YP decreased markedly, consistent with backbone scission, hydrolysis of amide groups, and partial disruption of bonds between the organic and inorganic phases. At every test temperature, SiO_2_@P-DAS provided better rheology and filtration control than P-DAS, indicating that the incorporation of modified nano-SiO_2_ contributes to improved temperature resistance.

**Figure 5:**
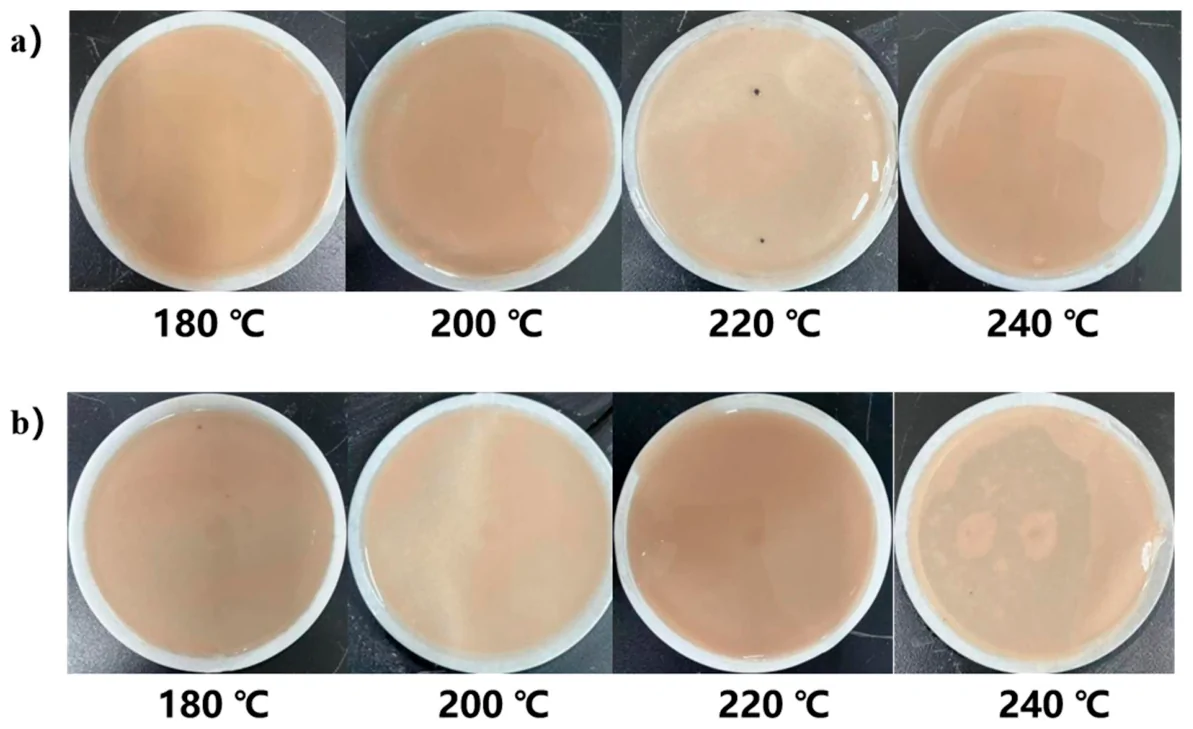
Filter cakes formed after aging P-DAS- and SiO_2_@P-DAS-treated drilling fluids for 16 h at different temperatures: (**a**) P-DAS; (**b**) SiO_2_@P-DAS.

As shown in Figure 5, across the aging range of 180–240 °C, the drilling fluid containing 3 wt% SiO_2_@P-DAS formed generally intact and compact filter cakes. With increasing temperature, only fine surface lines developed, and no structural failure was observed. These macroscopic characteristics indicate that SiO_2_@P-DAS retains strong high-temperature film-forming capability up to 220 °C and can meet deep-well requirements for filter-cake quality.

#### 2.2.3. NaCl and CaCl_2_ Resistance of the Fluid-Loss Reducers

To evaluate the resistance of P-DAS and SiO_2_@P-DAS to monovalent and divalent salt contamination, NaCl or CaCl_2_ was added at different concentrations to bentonite slurries containing 3.0 wt% additive. The drilling fluid performance under different NaCl dosages is shown in Table 1 and Table 2. The rheological parameters and API filtrate volumes were measured before and after aging at 220 °C for 16 h. As the NaCl concentration increased, the post-aging API filtrate volume of the P-DAS system increased from 5.6 to 9.8 mL, while its YP remained only 0.5–1.0 Pa at NaCl concentrations of 5–35 wt%, indicating a substantial loss of structural strength. In comparison, the post-aging AV, PV, and YP of the SiO_2_@P-DAS system remained within 21.0–27.0 mPa·s, 18.0–24.0 mPa·s, and 3.0–4.5 Pa, respectively. Its API filtrate volume increased from 3.5 to 7.3 mL but remained lower than that of P-DAS at every NaCl concentration. The drilling fluid performance under different CaCl_2_ dosages is shown in Table 3 and Table 4. CaCl_2_ produced a more pronounced deterioration because divalent Ca^2+^ ions more strongly compress the electrical double layer and promote clay-particle aggregation. As the CaCl_2_ content increased from 0 to 5 wt%, the post-aging API filtrate volume increased from 5.6 to 16.2 mL for P-DAS but only from 3.8 to 6.8 mL for SiO_2_@P-DAS. At 5 wt% CaCl_2_, SiO_2_@P-DAS reduced the post-aging filtrate volume by 58.0% relative to P-DAS and retained higher AV and PV values of 27 and 19 mPa·s, respectively, compared with 17.25 and 8 mPa·s for P-DAS. The improved resistance to both NaCl and CaCl_2_ may result from the strong hydration of the sulfonate groups, polymer adsorption on clay surfaces, and the reinforcing and particle-packing contributions of modified nano-SiO_2_. These effects may limit salt-induced particle aggregation and filter-cake deterioration, enabling SiO_2_@P-DAS to maintain better filtration control under high-temperature salt contamination.

#### 2.2.4. Drilling-Fluid Performance at Different Densities

The adaptability of the fluid-loss reducer to weighted drilling fluids was evaluated at densities ranging from 1.4 to 2.0 g/cm^3^. The results are shown in Table 5. Before aging, AV, PV, and YP generally increased with drilling-fluid density because of the higher solids content. After aging at 220 °C for 16 h, the rheological parameters varied with density, while the API filtrate volume remained low, decreasing from 3.2 mL at 1.4 g/cm^3^ to 2.4 mL at 2.0 g/cm^3^. The corresponding HTHP filtrate volume decreased slightly from 26.2 to 24.6 mL. These results indicate that the system maintains effective filtration control over the tested density range. The higher solids content may improve particle packing and promote the formation of a denser filter cake. However, at 2.0 g/cm^3^, the post-aging AV and PV increased to 86 and 76 mPa·s, respectively, indicating that the enhanced filtration control at high density must be balanced against the accompanying increase in viscosity.

### 2.3. Mechanistic Investigation

#### 2.3.1. Zeta-Potential Analysis

**Figure 6:**
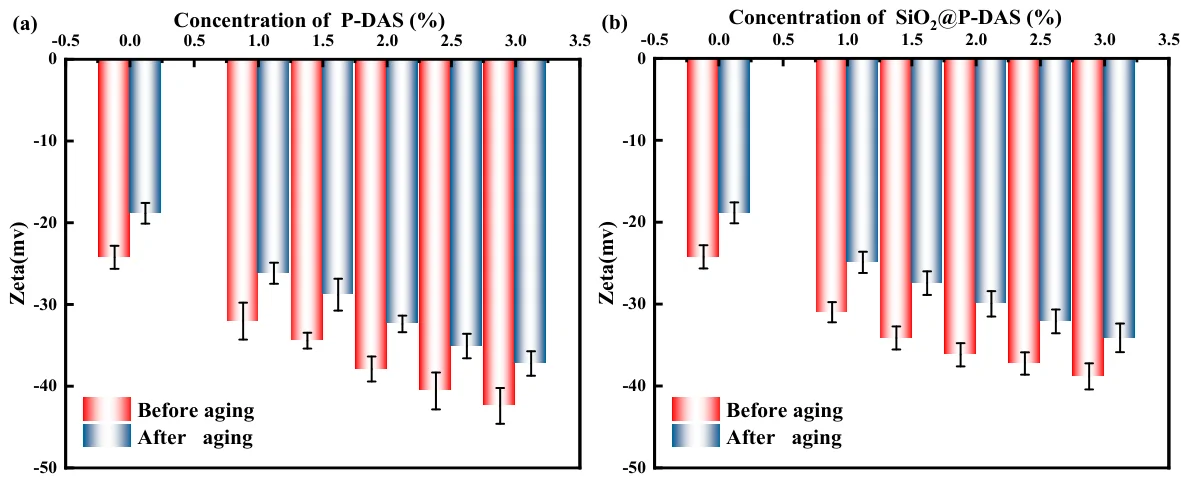
Zeta potentials of drilling fluids treated with different dosages of (**a**) P-DAS and (**b**) SiO_2_@P-DAS.

As the additive dosage increased from 1.0 to 4.0 wt%, the zeta potentials of both P-DAS and SiO_2_@P-DAS systems became progressively more negative. The test results are shown in Figure 6. Before aging, the potential changed from −32.04 to −44.57 mV for P-DAS and from −42.24 to −54.43 mV for SiO_2_@P-DAS. After aging at 220 °C for 16 h, the corresponding ranges were −26.03 to −40.27 mV and −40.13 to −52.70 mV. Increasing additive dosage therefore raised the absolute zeta potential of clay particles, strengthening electrostatic repulsion and promoting dispersion stability. At the same dosage, the absolute potential of SiO_2_@P-DAS was consistently greater than that of P-DAS both before and after aging. Aging reduced the absolute potential by an average of approximately 5.51 mV for P-DAS but only 1.93 mV for SiO_2_@P-DAS. At 3.0 wt%, the P-DAS potential changed from −40.28 to −35.54 mV, whereas the SiO_2_@P-DAS value changed from −51.47 to −48.37 mV. Nano-SiO_2_ therefore helps preserve the electrostatic-regulation capability of the composite additive during high-temperature aging, allowing clay particles to retain a strongly negative surface potential. Together with the filtration and filter-cake observations, this finding suggests that SiO_2_@P-DAS forms a relatively stable adsorption layer on clay surfaces and maintains electrostatic repulsion between particles.

As shown in Table 6, the zeta potential of a 4 wt% bentonite slurry containing 35 wt% NaCl was −24.1 mV at 25 °C and −18.5 mV after aging at 220 °C for 16 h, indicating that thermal aging further weakened electrostatic repulsion between clay particles under high salinity. Addition of 3 wt% P-DAS reduced the potential to −35.2 mV at 25 °C, but the value became −24.6 mV after aging; the 10.6 mV loss in absolute potential demonstrates marked attenuation of electrostatic regulation. In contrast, the potentials of the system containing 3 wt% SiO_2_@P-DAS were −48.3 mV at 25 °C and −45.8 mV after aging, a decrease of only 2.5 mV (5.2%). After aging, the absolute potential of the SiO_2_@P-DAS system exceeded those of the high-salinity base slurry and the P-DAS system by 27.3 and 21.2 mV, respectively. Thus, SiO_2_@P-DAS maintains a strongly negative clay-surface potential under high-temperature, high-salinity conditions and limits thermally induced electrostatic deterioration, which benefits dispersion stability. This may reflect the greater high-temperature stability of the polymer adsorption layer conferred by the nano-SiO_2_ composite structure.

#### 2.3.2. Particle-Size Analysis

**Figure 7:**
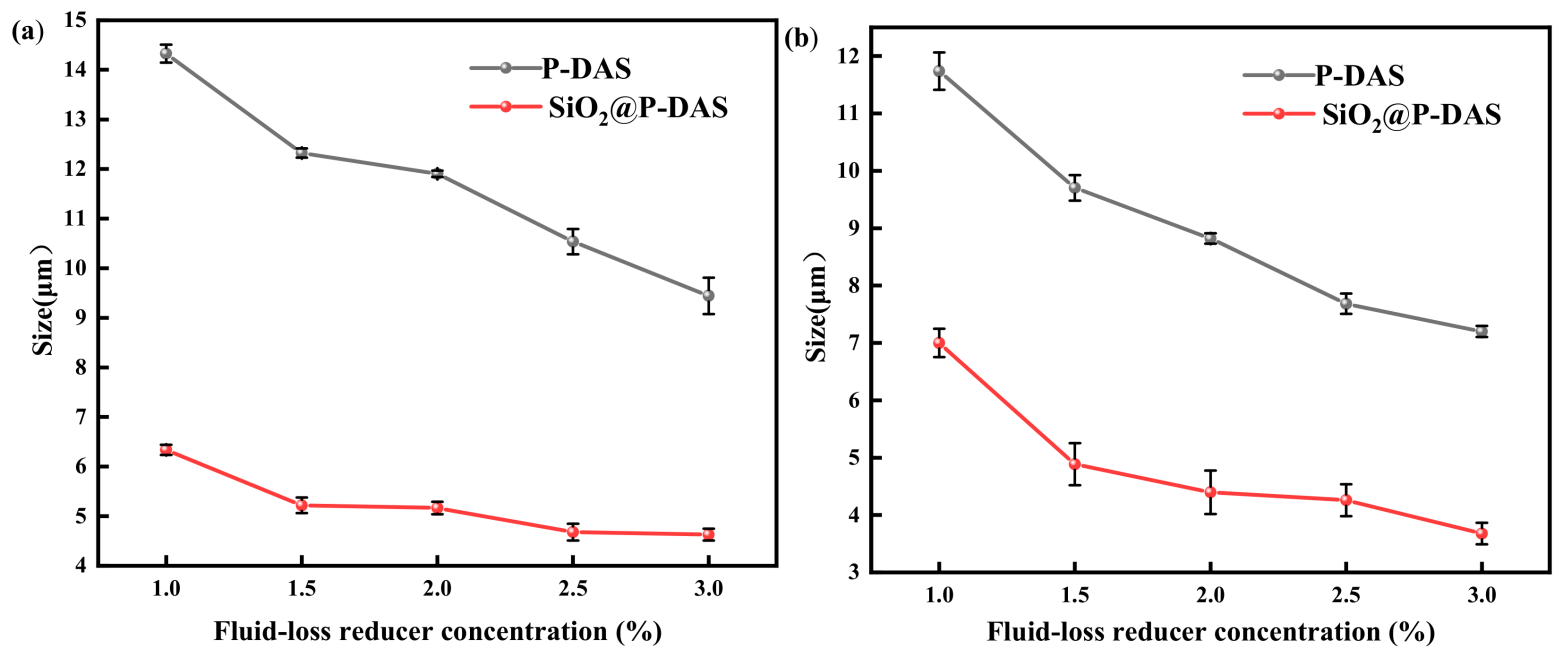
Particle-size distributions of drilling fluids containing different concentrations of P-DAS and SiO_2_@P-DAS: (**a**) before aging; (**b**) after aging.

As shown in Figure 7, as the additive concentration increased from 1.0 to 4.0 wt%, the Dx(50) of the P-DAS system generally decreased from 14.15 to 8.14 μm before aging and from 11.76 to 5.45 μm after aging. In contrast, the pre-aging Dx(50) of the SiO_2_@P-DAS system exhibited a non-monotonic variation within the range of 5.08–6.92 μm, while its post-aging value showed an overall decrease from 6.72 to 3.19 μm. The non-monotonic pre-aging variation may reflect the competing effects of polymer adsorption, particle bridging, and nanoparticle-assisted dispersion at different additive concentrations. Nevertheless, under the same dosage and aging conditions, SiO_2_@P-DAS generally produced a lower Dx(50) than P-DAS. At 3.0 wt%, the pre-aging Dx(50) values were 9.42 and 6.51 μm for P-DAS and SiO_2_@P-DAS, respectively, while the corresponding post-aging values were 7.26 and 4.88 μm. These results indicate that SiO_2_@P-DAS provides more effective control of the median particle size and limits the formation of large particle aggregates.

#### 2.3.3. Scanning Electron Microscopy

Filter-cake microstructures were compared by SEM for four systems: bentonite base slurry, salt-contaminated bentonite slurry, bentonite slurry containing SiO_2_@P-DAS, and salt-contaminated bentonite slurry containing SiO_2_@P-DAS. Filter cakes were prepared from each drilling fluid and examined by SEM.

**Figure 8:**
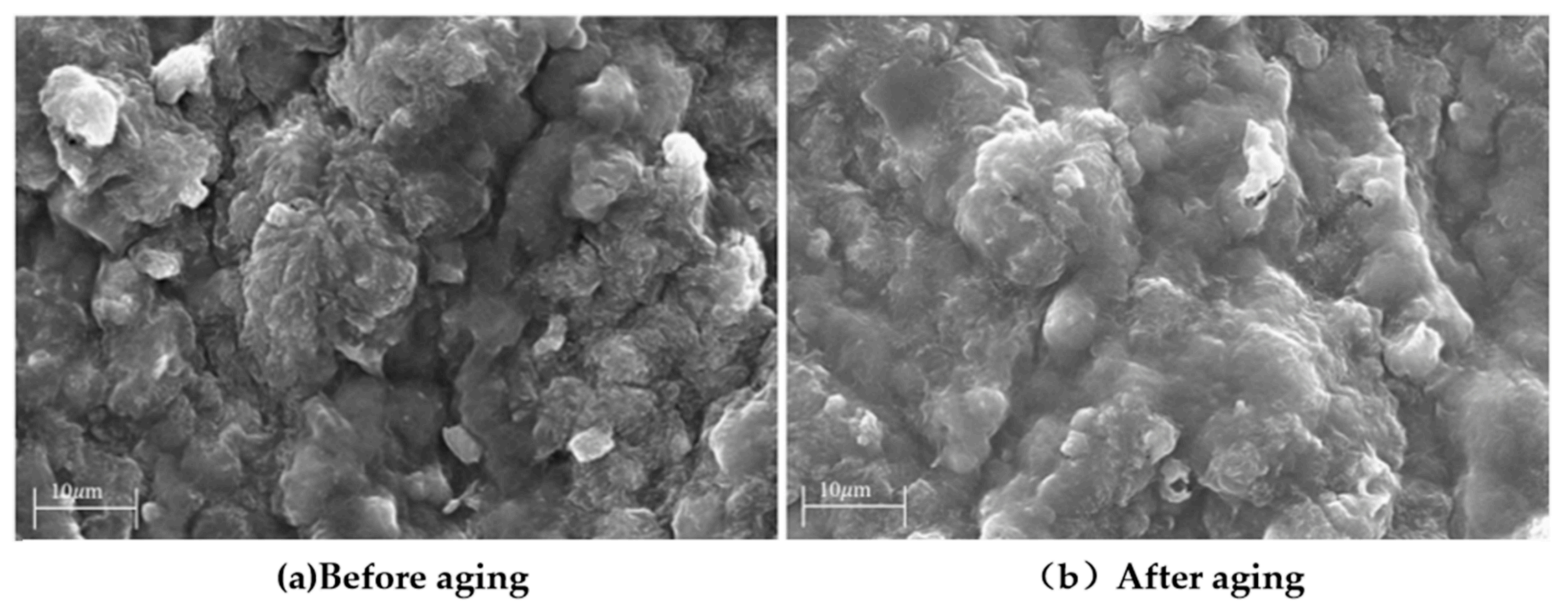
SEM images of bentonite-slurry filter cakes before and after aging.

**Figure 9:**
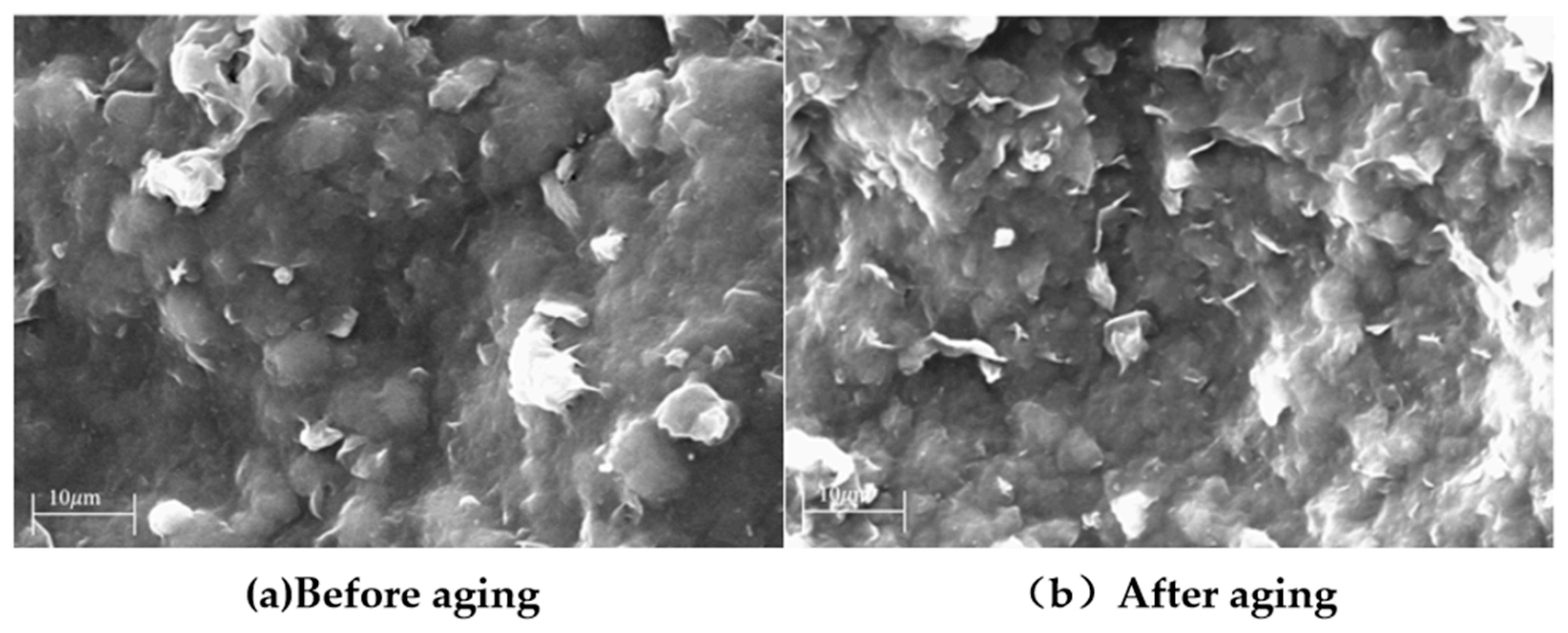
SEM images of filter cakes from bentonite slurry containing the fluid-loss reducer before and after aging.

As shown in Figure 8, particles in the unaged filter cake formed by the 4 wt% bentonite base slurry were loosely arranged; the surface was rough and contained numerous pores and fissures. After aging at 220 °C for 16 h, the platy clay particles aggregated into large clusters. Surface roughness and pore connectivity increased, and large voids and through-cracks developed, indicating substantial damage to filter-cake integrity. As shown in Figure 9, addition of SiO_2_@P-DAS produced a smoother surface, markedly fewer large pores and cracks, and more closely packed particles. After high-temperature aging, surface roughness increased somewhat, but the overall structure remained comparatively intact, with no obvious large aggregates or through-cracks and with smaller, more uniformly distributed pores. Based on the structure of SiO_2_@P-DAS, the polymer segments may regulate particle arrangement through adsorption and bridging, while nano-SiO_2_ participates in particle-size gradation and partly fills filter-cake pores, favoring formation of a relatively compact structure.

**Figure 10:**
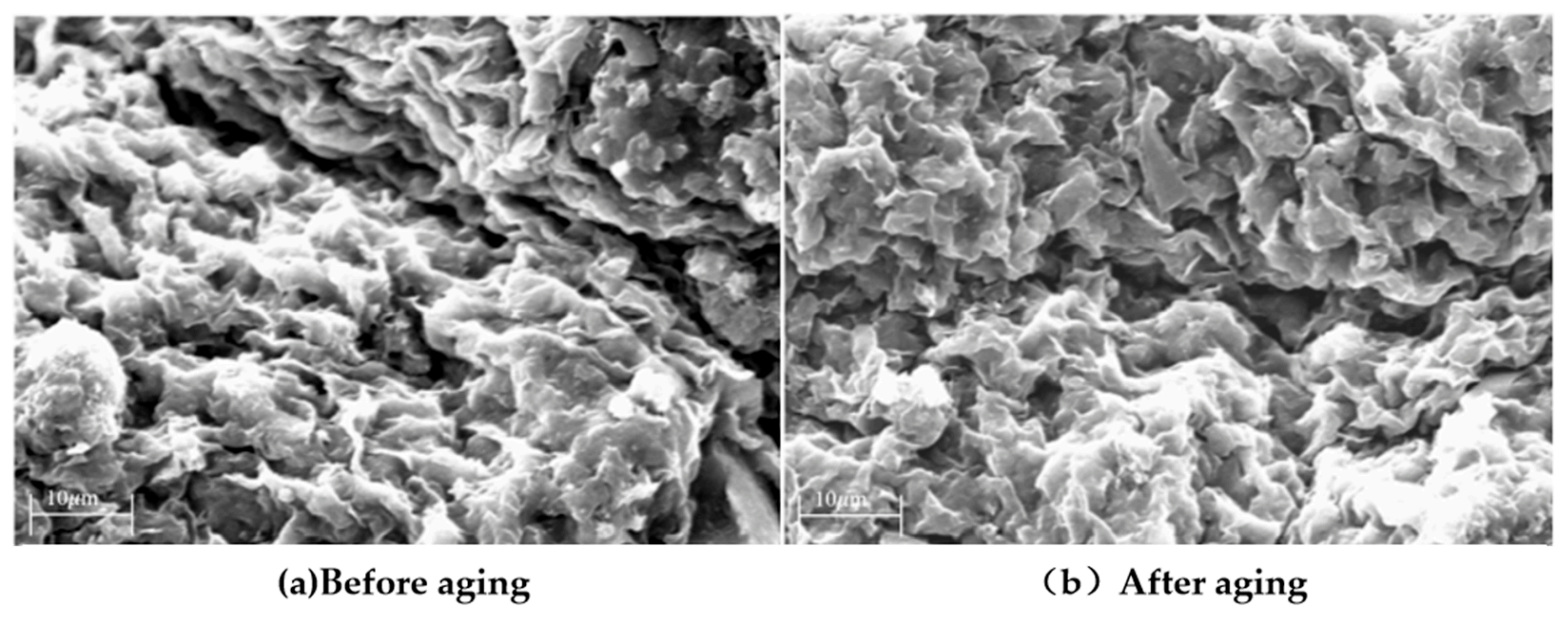
SEM images of filter cakes from salt-contaminated bentonite slurry before and after aging.

**Figure 11:**
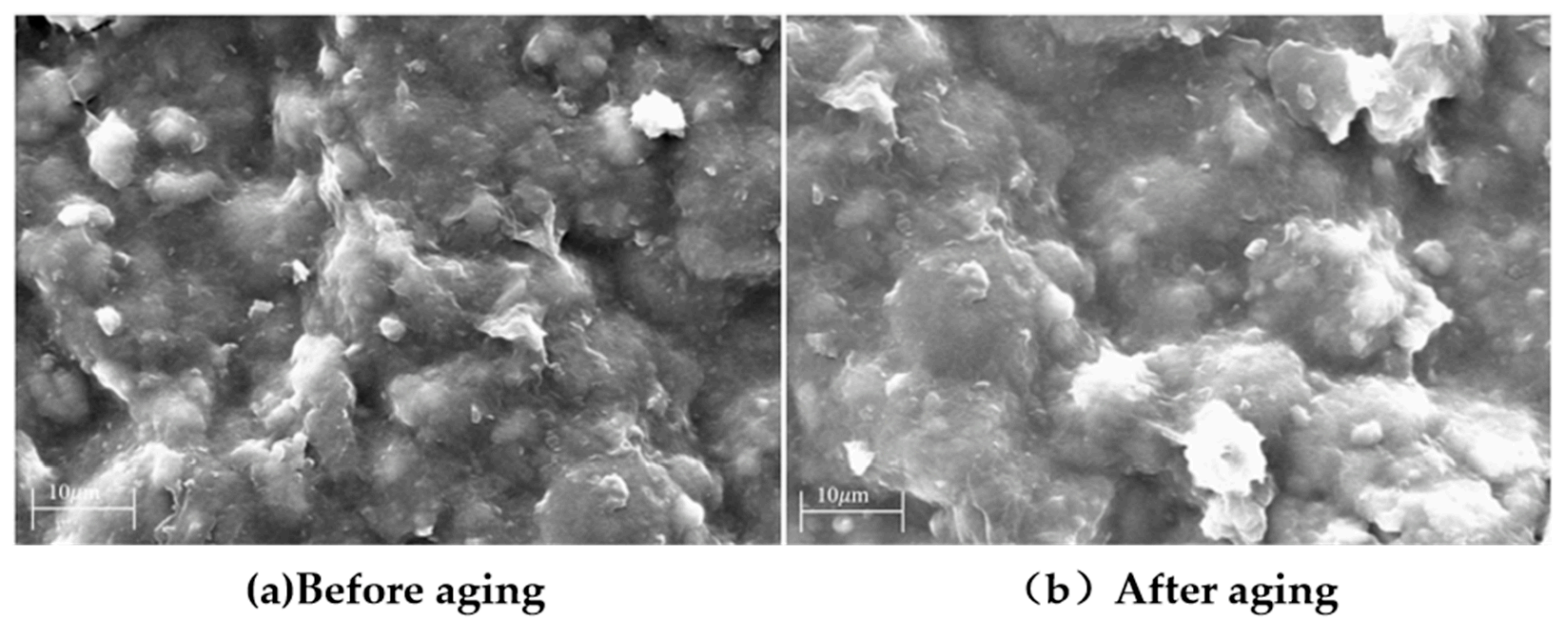
SEM images of filter cakes from salt-contaminated bentonite slurry containing the fluid-loss reducer before and after aging.

SEM images of the salt-contaminated bentonite base slurry and the salt-contaminated bentonite slurry containing SiO_2_@P-DAS are shown in Figure 10 and Figure 11.

In the salt-contaminated drilling fluid without a fluid-loss reducer, compression of the diffuse electrical double layer by salt ions likely weakened electrostatic repulsion and promoted clay aggregation. SEM images show a disordered particle arrangement, an uneven surface, numerous pores and fissures, high pore connectivity, and a loose overall structure. High-temperature aging intensified clay aggregation, increased surface roughness, and generated large voids and interconnected fissures, indicating that temperature and salinity jointly impaired filter-cake integrity. After addition of SiO_2_@P-DAS, the salt-containing filter cake was slightly rougher than its salt-free counterpart and contained a small number of fine aggregates, but large pores and cracks were markedly reduced and the overall structure remained relatively intact and compact. Following high-temperature aging, surface roughness and the number of small pores increased, yet no obvious large voids or through-cracks were observed, and the main structure remained intact. Together with the zeta-potential and particle-size results, these observations suggest that adsorption by the polymer segments regulates interactions between clay particles, while nano-SiO_2_ contributes to particle-size gradation and pore-structure adjustment. These effects limit excessive aggregation and filter-cake deterioration under high-temperature, high-salinity conditions and improve structural retention.

#### 2.3.4. Mechanism Analysis

**Figure 12:**
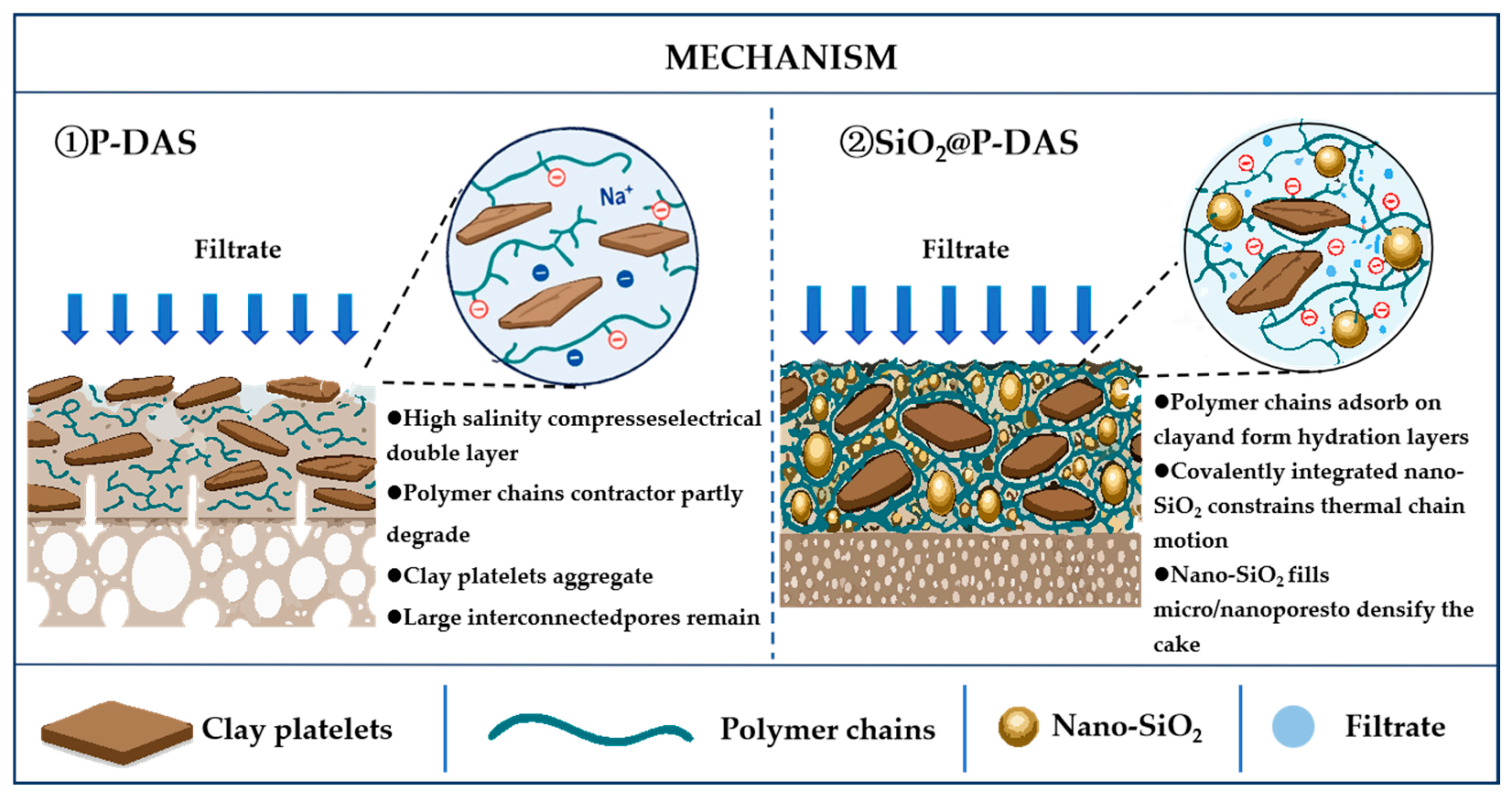
Mechanism Analysis.

As shown in Figure 12, SiO_2_@P-DAS improves the filtration-control performance of high-temperature, high-salinity water-based drilling fluids through the synergistic effects of polymer adsorption and hydration, rigid nanofiller reinforcement, electrostatic stabilization, and micro-/nanopore filling. The amide and sulfonate groups in P-DAS adsorb onto clay-particle surfaces and form protective hydration layers. Under high-temperature and high-salinity conditions, however, salt ions compress the diffuse electrical double layer, while the polymer chains contract or partially degrade. Consequently, electrostatic repulsion and steric hindrance between clay particles are weakened, promoting particle aggregation and the formation of a loose filter cake with interconnected pores. After the introduction of KH-570-modified nano-SiO_2_, the inorganic component may act as a rigid reinforcing domain within the composite, restricting polymer-segment motion and retarding thermally induced structural degradation. The adsorption of SiO_2_@P-DAS onto clay surfaces helps maintain a relatively high negative surface potential and a stable hydration layer, thereby enhancing electrostatic repulsion and steric stabilization between particles. Meanwhile, nano-SiO_2_ and the stably dispersed fine particles may enter small pores between clay platelets, improve the particle-size gradation of the solids, and contribute to reduced pore connectivity within the filter cake. Particle-size analysis and microscopic observations further indicate that the SiO_2_@P-DAS system exhibits less particle aggregation and forms a more continuous, compact, and low-permeability filter cake. These multiscale synergistic effects increase the tortuosity and resistance of filtrate-flow pathways, enabling SiO_2_@P-DAS to maintain favorable rheological stability and filtration control under high-temperature and high-salinity conditions. The proposed mechanism is supported by the combined filtration, zeta-potential, particle-size, and SEM results. The higher absolute zeta potential and lower Dx(50) indicate improved particle dispersion, while the SEM observations show fewer large pores and through-cracks in the SiO_2_@P-DAS-treated filter cakes. Together with the lower API and HTHP filtrate volumes, these findings suggest that modified nano-SiO_2_ may contribute to improved particle packing, filter-cake continuity, and filtration control.

## 3. Conclusions

(1) SiO_2_@P-DAS was synthesized through aqueous free-radical polymerization in the presence of KH-570-modified nano-SiO_2_, with P-DAS used as the control. FTIR results were consistent with the presence of modified nano-SiO_2_ in the composite. The initial decomposition temperature increased from 245 to 252 °C, indicating improved thermal stability.

(2) Based on the combined rheological and filtration results, 3.0 wt% was selected as the working concentration. After aging at 220 °C for 16 h, the API filtrate volumes of SiO_2_@P-DAS and P-DAS were 3.8 and 4.8 mL, respectively. Their HTHP filtrate volumes at 220 °C and 3.5 MPa were 33.33 and 49.67 mL, confirming the improved filtration-control performance of SiO_2_@P-DAS.

(3) SiO_2_@P-DAS maintained better filtration control than P-DAS under both NaCl and CaCl_2_ contamination. At 5 wt% CaCl_2_, its post-aging API filtrate volume was 6.8 mL, compared with 16.2 mL for P-DAS. In weighted drilling fluids with densities ranging from 1.4 to 2.0 g/cm^3^, the post-aging API and HTHP filtrate volumes remained within 2.4–3.2 and 24.6–26.2 mL, respectively. However, the increased AV and PV at 2.0 g/cm^3^ indicate that filtration control must be balanced against viscosity at high density.

(4) The zeta-potential, particle-size, and filter-cake results suggest that SiO_2_@P-DAS improves particle dispersion and filter-cake continuity. These effects may arise from polymer adsorption and hydration, together with the contribution of modified nano-SiO_2_ to particle packing and structural reinforcement. Under the present 16 h aging protocol, SiO_2_@P-DAS maintained favorable performance up to 220 °C, whereas evident deterioration occurred at 240 °C. Further evaluation under longer aging periods and mixed formation-brine conditions is still required.

## 4. Materials and Methods

### 4.1. Materials and Instruments

N,N-Dimethylacrylamide (DMAA) and sodium styrene sulfonate (SSS) were purchased from Shanghai Macklin Biochemical Technology Co., Ltd. (Shanghai, China), 2-Acrylamido-2-methylpropane sulfonic acid (AMPS) was obtained from Saan Chemical Technology (Shanghai) Co., Ltd. (Shanghai, China), and KH-570-modified silica was supplied by Jiangsu Xianfeng Nanomaterials Technology Co., Ltd. (Nanjing, China). Additionally, 2,2′-Azobis(2-methylpropionamidine) dihydrochloride (V50), sodium chloride (NaCl), and sodium hydroxide (NaOH) were purchased from Tianjin Zhonglian Chemical Reagent Co., Ltd. (Tianjin, China). Anhydrous sodium carbonate (Na_2_CO_3_) was obtained from Shanghai Macklin Biochemical Technology Co., Ltd. (Shanghai, China), and the defoamer was supplied by Jiangsu Xianfeng Nanomaterials Technology Co., Ltd. (Nanjing, China).

The instruments used were a Sigma 300 scanning electron microscope (Carl Zeiss, Oberkochen, Germany), a Thermo Nicolet Fourier-transform infrared spectrometer (Thermo Fisher Scientific, Waltham, MA, USA), a TGA2 thermogravimetric analyzer (Mettler Toledo, Greifensee, Switzerland), a BLF multifunctional pulverizer (Shanghai Bilang, Shanghai, China), a Mastersizer laser particle-size analyzer and a Zetasizer Nano zeta-potential analyzer (Malvern Panalytical, Malvern, UK), a BRGL-7 roller oven and a high-speed variable-frequency mixer (Qingdao Tongchun, Qingdao, China), an HH-4 digital thermostatic water bath (Shanghai Yiheng, Shanghai, China), a six-speed rotational viscometer (Qingdao Haitongda, Qingdao, China), a DF-101S thermostatic magnetic stirrer (Zhengzhou Ketai, Zhengzhou, China), a forced-air drying oven (Shanghai Longyue, Shanghai, China), an API filter press (Qingdao Tongchun, Qingdao, China), and an electronic balance (Mettler Toledo, Shanghai, China).

### 4.2. Molecular Design of the Fluid-Loss Reducer

Because all-organic polymers may undergo chain degradation, conformational contraction, and loss of adsorption capacity under high-temperature, high-salinity conditions, an organic–inorganic nanocomposite gel fluid-loss reducer was designed. The amide carbonyl groups in DMAA enhance polymer hydration and interfacial interactions with clay surfaces. The sulfonic acid groups in AMPS provide strong hydration and good brine compatibility, whereas SSS combines hydrophilic sulfonate groups with a rigid aromatic structure to enhance salt resistance and thermal stability. KH-570 is anchored to the nano-SiO_2_ surface through siloxane bonds; its unsaturated double bond then participates in free-radical copolymerization, covalently incorporating the nanoparticles into the polymer. The resulting rigid nano-SiO_2_ nodes are expected to restrict excessive segmental motion at elevated temperature, retard structural degradation, improve particle-size gradation in the filter cake through nanoscale filling, and reduce interconnected pores. The intended mechanism is thus a synergy between polymer-chain adsorption/hydration and the rigid reinforcement and pore-filling functions of nano-SiO_2_, providing structural stability and sustained filtration control in high-temperature, high-salinity water-based drilling fluids.

### 4.3. Synthesis of the Nanocomposite Fluid-Loss Reducer

The mass ratio of DMAA, AMPS, SSS, and KH-570-modified nano-SiO_2_ was 10:14:10:1, and the total monomer concentration was 30 wt%. AMPS was first dissolved in deionized water, and the pH of the solution was adjusted to 7–8 using a 30 wt% NaOH solution. DMAA and SSS were then added sequentially and dissolved under continuous stirring. Subsequently, KH-570-modified nano-SiO_2_, corresponding to 3 wt% of the total monomer mass, was added and dispersed by continuous stirring.

The resulting mixture was transferred to a three-neck round-bottom flask equipped with a mechanical stirrer and purged with nitrogen for 30 min. The reaction mixture was then heated to 60 °C in a water bath while being stirred at 350 rpm. V50, at a dosage corresponding to 1 wt% of the total monomer mass, was added dropwise, and the polymerization was allowed to proceed at 60 °C for 5 h. The resulting gel product was transferred directly to a tray without further purification, dried in an oven at 80 ± 1 °C for 24 h, and subsequently pulverized to obtain SiO_2_@P-DAS.

P-DAS was prepared as the nano-free control using the same monomer ratio, total monomer concentration, pH, reaction temperature, stirring rate, initiator dosage, reaction time, and drying procedure, but without the addition of KH-570-modified nano-SiO_2_. Because the products were dried and used directly without isolation or purification of individual fractions, the isolated yield, monomer conversion, and grafting efficiency were not determined in this study.

### 4.4. Characterization of the Fluid-Loss Reducers

1. Fourier-transform infrared spectroscopy (FTIR): SiO_2_@P-DAS and P-DAS were separately mixed with potassium bromide (KBr) and pressed into translucent pellets. FTIR spectra were recorded over 4000–400 cm^−1^.

2. Thermogravimetric analysis (TGA): Approximately 8 mg of SiO_2_@P-DAS or P-DAS was placed in a crucible and analyzed under nitrogen from 25 to 600 °C at a heating rate of 5 °C/min.

### 4.5. Evaluation of Rheological and Filtration Properties

A base drilling-fluid slurry was prepared by adding 40 g of drilling-fluid-grade bentonite and 3.0 g of anhydrous sodium carbonate to 1000 mL of water, followed by mixing at 8000 rpm for 20 min. The slurry was sealed and hydrated at room temperature for 24 h. The slurry was sealed and hydrated at room temperature for 24 h. Rheological properties were measured with a six-speed rotational viscometer, filtrate volume with an API filter press, and thermal aging for 16 h at the specified temperatures in a roller oven.

1. Different concentrations of SiO_2_@P-DAS or P-DAS were added to the base slurry and mixed for 20 min. The rheological parameters and API filtrate volumes were measured before and after hot-rolling aging at 220 °C for 16 h. For the HTHP filtration tests, the aged drilling fluids were thoroughly remixed, transferred to an HTHP filtration cell, and heated to 220 °C. After the temperature had stabilized, a differential pressure of 3.5 MPa was applied, and the filtrate passing through a stainless-steel sintered filter disk was collected for 30 min. The cumulative filtrate volume was recorded as the HTHP filtrate volume. Each measurement was performed in triplicate.

The rheological properties were measured using a six-speed rotational viscometer. After thorough mixing, the drilling fluid was transferred to the sample cup and the dial readings at 600 and 300 rpm were recorded. Each measurement was performed in triplicate. The rheological parameters were calculated using Equations (1)–(3).

(1) *PV* = Φ_600_ − Φ_300_

(2) *YP* = 0.511 × (Φ_300_ − *PV*)

(3) *AV* = 0.5 × Φ_600_

where *PV* is the plastic viscosity (mPa·s), *YP* is the yield point (Pa), *AV* is the apparent viscosity (mPa·s). Φ_600_ and Φ_300_ are the viscometer dial readings at 600 and 300 rpm, respectively, and the factor 0.511 converts the yield-point value from lb/100 ft^2^ to Pa.

2. Temperature-resistance evaluation: SiO_2_@P-DAS or P-DAS was added to the base slurry at 3 wt% and mixed for 20 min. The drilling fluids were then aged for 16 h at different temperatures, after which their rheological and filtration properties were measured.

3. Salt-resistance evaluation: SiO_2_@P-DAS or P-DAS (3.0 wt%) and different concentrations of NaCl or CaCl_2_ were added separately to the base slurry. The rheological parameters and API filtrate volumes were measured before and after aging at 220 °C for 16 h.

### 4.6. Mechanistic Investigation

1. Zeta-potential analysis: Different concentrations of SiO_2_@P-DAS or P-DAS were added to the base slurry. Zeta potentials were measured before and after aging at 220 °C.

2. Particle-size analysis: SiO_2_@P-DAS or P-DAS was added at 3 wt% to saturated-brine base slurry. Changes in particle-size distribution before and after aging were measured using a laser particle-size analyzer.

3. Microscopic filter-cake morphology: Base slurry, drilling fluids containing 3 wt% SiO_2_@P-DAS or P-DAS, and saturated-brine drilling fluids containing 3 wt% SiO_2_@P-DAS or P-DAS were aged at 220 °C for 16 h. Filter cakes were obtained from API filtration tests, dried, and examined by scanning electron microscopy (SEM).

## Figures and Tables

**Table 1:** NaCl resistance of drilling fluid 1 before and after aging at 220 °C for 16 h.

Formulation	Aging Condition	AV (mPa s)	PV (mPa s)	YP (Pa)	API Filtrate (mL)
Fluid 1 + 0% NaCl	Before aging	80	51	29	4.8
	After aging at 220 °C	60	40	20	5.6
Fluid 1 + 5% NaCl	Before aging	15	8	7	5.2
	After aging at 220 °C	8.5	8.0	0.5	6.4
Fluid 1 + 15% NaCl	Before aging	16.5	7.5	9	5.8
	After aging at 220 °C	9.0	8.0	1.0	6.9
Fluid 1 + 25% NaCl	Before aging	18	8.5	9.5	6.8
	After aging at 220 °C	10.5	10	0.5	8.4
Fluid 1 + 35% NaCl	Before aging	20	12	8	7.5
	After aging at 220 °C	12.0	11.0	1.0	9.8

**Table 2:** NaCl resistance of drilling fluid 2 before and after aging at 220 °C for 16 h.

Formulation	Test Condition	AV (mPa s)	PV (mPa s)	YP (Pa)	API Filtrate (mL)
Fluid 2 + 0% NaCl	Before aging	69.5	48	21.5	2.6
	After aging at 220 °C	52	36	16	3.8
Fluid 2 + 5% NaCl	Before aging	50	20	30	3.2
	After aging at 220 °C	21	18	3	3.5
Fluid 2 + 15% NaCl	Before aging	62.5	25	37.5	3.8
	After aging at 220 °C	24	20	4	4.7
Fluid 2 + 25% NaCl	Before aging	68	26	42	4.6
	After aging at 220 °C	24.5	20	4.5	5.7
Fluid 2 + 35% NaCl	Before aging	76.5	34	42.5	5.2
	After aging at 220 °C	27	24	3	7.3

**Table 3:** CaCl_2_ resistance of drilling fluid 1 before and after aging at 220 °C for 16 h.

Formulation	Test Condition	AV (mPa s)	PV (mPa s)	YP (Pa)	API Filtrate (mL)
Fluid 1 + 0% CaCl_2_	Before aging	80.00	51.00	29.64	4.8
	After aging at 220 °C	60.00	40.00	20.44	5.6
Fluid 1 + 1% CaCl_2_	Before aging	49.00	31.00	18.40	6.8
	After aging at 220 °C	7.50	4.00	3.58	7.8
Fluid 1 + 2% CaCl_2_	Before aging	40.00	25.00	15.33	8.2
	After aging at 220 °C	10.00	6.00	4.09	9.8
Fluid 1 + 3% CaCl_2_	Before aging	33.75	21.50	12.52	9.6
	After aging at 220 °C	12.25	6.00	6.39	11.8
Fluid 1 + 4% CaCl_2_	Before aging	27.25	17.50	9.96	11.2
	After aging at 220 °C	14.75	7.50	7.41	13.8
Fluid 1 + 5% CaCl_2_	Before aging	21.00	14.50	6.64	12.6
	After aging at 220 °C	17.25	8.00	9.45	16.2

**Table 4:** CaCl_2_ resistance of drilling fluid 2 before and after aging at 220 °C for 16 h.

Formulation	Test Condition	AV (mPa s)	PV (mPa s)	YP (Pa)	API Filtrate (mL)
Fluid 2 + 0% CaCl_2_	Before aging	69.5	48	21.5	2.6
	After aging at 220 °C	52	36	16	3.8
Fluid 2 + 1% CaCl_2_	Before aging	37	27	10	3.4
	After aging at 220 °C	8	4	4	4.8
Fluid 2 + 2% CaCl_2_	Before aging	32.5	23	9.5	3.8
	After aging at 220 °C	13	8	5	5.2
Fluid 2 + 3% CaCl_2_	Before aging	31.5	23	8.5	4.2
	After aging at 220 °C	12	10	2	5.8
Fluid 2 + 4% CaCl_2_	Before aging	31	22	9	4.6
	After aging at 220 °C	15	12	3	6.2
Fluid 2 + 5% CaCl_2_	Before aging	30.5	22	8.5	5.2
	After aging at 220 °C	27	19	8	6.8

Drilling fluid 1: 4.0 wt% bentonite slurry + 3.0 wt% P-DAS. Drilling fluid 2: 4.0 wt% bentonite slurry + 3.0 wt% SiO_2_@P-DAS.

**Table 5:** Rheological and filtration properties of SiO_2_@P-DAS-treated drilling fluids at different densities before and after aging at 220 °C for 16 h.

Drilling Fluid Density	Aging Condition	AV (mPa s)	PV (mPa s)	YP (Pa)	API Filtrate (mL)	FL_HTHP_ (mL)
1.4 g/cm^3^	Before aging	36.00	26.00	10.22	2	26.2
	After aging at 220 °C	26.00	20.00	6.13	3.2	
1.6 g/cm^3^	Before aging	50.00	38.00	12.26	1.9	25.4
	After aging at 220 °C	40.00	36.00	4.09	2.8	
1.8 g/cm^3^	Before aging	72.00	58.00	14.31	1.6	25.2
	After aging at 220 °C	38.00	34.00	4.09	2.7	
2.0 g/cm^3^	Before aging	94.00	64.00	30.66	1.4	24.6
	After aging at 220 °C	86.00	76.00	10.22	2.4	

**Table 6:** Zeta potentials of NaCl-contaminated fluids containing SiO_2_@P-DAS or P-DAS.

Drilling Fluid	Test Condition	Zeta Potential (mV)
4% bentonite slurry + 35% NaCl	25 °C After aging at 220 °C for 16 h	−24.1 −18.5
4% bentonite slurry + 3% P-DAS + 35% NaCl	25 °C After aging at 220 °C for 16 h	−35.2 −24.6
4% bentonite slurry + 3% SiO_2_@P-DAS + 35% NaCl	25 °C After aging at 220 °C for 16 h	−48.3 −45.8

## Data Availability

The data presented in this study are available on request from the corresponding author.

## References

[1] Huang X., Sun J., Lyu K., Dong X., Liu F., Gao C. (2023). A High-Temperature Resistant and High-Density Polymeric Saturated Brine-Based Drilling Fluid. Pet. Explor. Dev..

[2] Thaemlitz C.J., Patel A.D., Coffin G., Conn L. (1999). New Environmentally Safe High-Temperature Water-Based Drilling-Fluid System. SPE Drill. Complet..

[3] Zhu W., Zheng X. (2021). Effective Modified Xanthan Gum Fluid Loss Agent for High-Temperature Water-Based Drilling Fluid and the Filtration Control Mechanism. ACS Omega.

[4] Li J., Ji Y.-X., Ni X.-X., Lv K.-H., Huang X.-B., Sun J.-S. (2024). A Micro-Crosslinked Amphoteric Hydrophobic Association Copolymer as High Temperature- and Salt-Resistance Fluid Loss Reducer for Water-Based Drilling Fluids. Pet. Sci..

[5] Liu F., Jiang G., Peng S., He Y., Wang J. (2016). Amphoteric Polymer as an Anti-Calcium Contamination Fluid-Loss Additive in Water-Based Drilling Fluids. Energy Fuels.

[6] Ma J., Xia B., Yu P., An Y. (2020). Comparison of an Emulsion- and Solution-Prepared Acrylamide/AMPS Copolymer for a Fluid Loss Agent in Drilling Fluid. ACS Omega.

[7] Li D., Bai Y., Liu L., Su N., Hu J., Wang C., Wu L., Wu H., Ma Y., Luo P. (2026). High-Temperature and High-Salt Water-Based Drilling Fluid Loss Reducing Agent: Synthesis, Performance Evaluation and Mechanism. Colloids Surf. A Physicochem. Eng. Asp..

[8] Quan H., Li H., Huang Z., Zhang T., Dai S. (2014). Copolymer SJ-1 as a Fluid Loss Additive for Drilling Fluid with High Content of Salt and Calcium. Int. J. Polym. Sci..

[9] Wan T., Yao J., Zishun S., Li W., Juan W. (2011). Solution and Drilling Fluid Properties of Water Soluble AM–AA–SSS Copolymers by Inverse Microemulsion. J. Pet. Sci. Eng..

[10] Wu Y.M., Sun D.J., Zhang B.Q., Zhang C.G. (2002). Properties of High-temperature Drilling Fluids Incorporating Disodium Itaconate/Acrylamide/Sodium 2-acrylamido-2-methylpropanesulfonate Terpolymers as Fluid-loss Reducers. J. Appl. Polym. Sci..

[11] Hamad B.A., He M., Xu M., Liu W., Mpelwa M., Tang S., Jin L., Song J. (2020). A Novel Amphoteric Polymer as a Rheology Enhancer and Fluid-Loss Control Agent for Water-Based Drilling Muds at Elevated Temperatures. ACS Omega.

[12] Wang R., Yang J., Liu L., Wang J., Feng Z., Zhang D., Gao S., Wang J., Ren H., Hui B. (2022). Investigation on Filtration Control of Zwitterionic Polymer AADN in High Temperature High Pressure Water-Based Drilling Fluids. Gels.

[13] Liu L., Sun J., Wang R., Liu F., Gao S., Yang J., Ren H., Qu Y., Cheng R., Geng Y. (2022). New Zwitterionic Polymer as a Highly Effective Salt- and Calcium-Resistant Fluid Loss Reducer in Water-Based Drilling Fluids. Gels.

[14] Shan W., Ma J., Jiang G., Sun J., An Y. (2022). An Inverse Emulsion Polymer as a Highly Effective Salt- and Calcium-Resistant Fluid Loss Reducer in Water-Based Drilling Fluids. ACS Omega.

[15] Kök M.V., Bal B. (2019). Effects of Silica Nanoparticles on the Performance of Water-Based Drilling Fluids. J. Pet. Sci. Eng..

[16] Yang J., Wang R., Sun J., Qu Y., Ren H., Zhao Z., Wang P., Li Y., Liu L. (2024). Comb Polymer/Layered Double Hydroxide (LDH) Composite as an Ultrahigh Temperature Filtration Reducer for Water-Based Drilling Fluids. Appl. Surf. Sci..

[17] Sun J., Chang X., Zhang F., Bai Y., Lv K., Wang J., Zhou X., Wang B. (2020). Salt-Responsive Zwitterionic Polymer Brush Based on Modified Silica Nanoparticles as a Fluid-Loss Additive in Water-Based Drilling Fluids. Energy Fuels.

[18] Li J., Sun J., Lv K., Ji Y., Ji J., Liu J. (2022). Nano-Modified Polymer Gels as Temperature- and Salt-Resistant Fluid-Loss Additive for Water-Based Drilling Fluids. Gels.

[19] Mao H., Qiu Z., Shen Z., Huang W. (2015). Hydrophobic Associated Polymer Based Silica Nanoparticles Composite with Core–Shell Structure as a Filtrate Reducer for Drilling Fluid at Utra-High Temperature. J. Pet. Sci. Eng..

[20] Dong X., Sun J., Huang X., Lv K., Zhou Z., Gao C. (2022). Nano-Laponite/Polymer Composite as Filtration Reducer on Water-Based Drilling Fluid and Mechanism Study. R. Soc. Open Sci..

[21] Hajiabadi S.H., Bedrikovetsky P., Mahani H., Khoshsima A., Aghaei H., Kalateh-Aghamohammadi M., Habibi S. (2020). Effects of Surface Modified Nanosilica on Drilling Fluid and Formation Damage. J. Pet. Sci. Eng..

[22] Xu Z., Sun J.-S., Liu J.-P., Lv K.-H., Dong X.-D., Wang Z.-L., Zhang T.-F., Sun Y.-W., Dai Z.-W. (2024). Development and Performance Evaluation of High Temperature Resistant Strong Adsorption Rigid Blocking Agent. Pet. Sci..

[23] Huang X.-B., Sun J.-S., Huang Y., Yan B.-C., Dong X.-D., Liu F., Wang R. (2021). Laponite: A Promising Nanomaterial to Formulate High-Performance Water-Based Drilling Fluids. Pet. Sci..

[24] Ahmed A., Pervaiz E., Abdullah U., Noor T. (2024). Optimization of Water Based Drilling Fluid Properties with the SiO_2_/g-C_3_N_4_ Hybrid. ACS Omega.

[25] Rafieefar A., Sharif F., Hashemi A., Bazargan A.M. (2021). Rheological Behavior and Filtration of Water-Based Drilling Fluids Containing Graphene Oxide: Experimental Measurement, Mechanistic Understanding, and Modeling. ACS Omega.

[26] Ma J., Pang S., Zhang Z., Xia B., An Y. (2021). Experimental Study on the Polymer/Graphene Oxide Composite as a Fluid Loss Agent for Water-Based Drilling Fluids. ACS Omega.

[27] Lin L., Luo Y. (2024). Polymer–SiO 2/Laponite Composite as a Filtrate Reducer for High-Temperature and High-Salt Drilling Fluids. ACS Appl. Nano Mater..

[28] Parizad A., Shahbazi K., Tanha A.A. (2018). SiO2 Nanoparticle and KCl Salt Effects on Filtration and Thixotropical Behavior of Polymeric Water Based Drilling Fluid: With Zeta Potential and Size Analysis. Results Phys..

[29] Oseh J.O., Mohd Norddin M.N.A., Ismail I., Gbadamosi A.O., Agi A., Mohammed H.N. (2019). A Novel Approach to Enhance Rheological and Filtration Properties of Water–Based Mud Using Polypropylene–Silica Nanocomposite. J. Pet. Sci. Eng..

[30] Li H., Sun J., Xie S., Lv K., Huang X., Zong J., Zhang Y. (2023). Controlling Filtration Loss of Water-Based Drilling Fluids by Anionic Copolymers with Cyclic Side Groups: High Temperature and Salt Contamination Conditions. Colloids Surf. A Physicochem. Eng. Asp..

[31] Tchameni A.P., Nagre R.D., Zhang S., Su J., Wang X., Wei Z. (2026). Tailored Water-Based Drilling Muds with Thermo-Responsive Polymer-Based Janus Biogenic Silica Nanocomposite for Filtration Loss Control in High-Temperature and High-Salinity Environments. Ind. Crops Prod..

[32] Luo Y., Lin L. (2026). Effects of Oxide Particle Size, Type, and Polymer Incorporation Methods on the Performance of Fluid Loss Additives for High-Temperature and High-Salinity Drilling Fluids. Energy Fuels.

